# Toxic Emissions from a Military Test Site in the Territory of Sardinia, Italy

**DOI:** 10.3390/ijerph10041631

**Published:** 2013-04-19

**Authors:** Mauro Cristaldi, Cristiano Foschi, Germana Szpunar, Carlo Brini, Fiorenzo Marinelli, Lucio Triolo

**Affiliations:** 1 Department of Biology and Biotechnologies “C. Darwin”, Sapienza University of Rome, via A. Borelli 50, Rome 00161, Italy; E-Mails: saiagio@libero.it (C.F.); germana.szpunar@gmail.com (G.S.); 2 Formerly with Veterinary Service of Local Health Agency, Biella 13900, Italy; E-Mail: carlo.brini@gmail.com; 3 Molecular Genetic Institute of Research National Council at I.O. Rizzoli, c/o Istituto Ortopedico Rizzoli-via di Barbiano 1/10, Bologna 40136, Italy; E-Mail: marinelli@area.bo.cnr.it; 4 Formerly with BAS-SIC (Environmental and Health Safety Lab.), ENEA-Casaccia, Rome 00123, Italy; E-Mail: lucio.triolo@fastwebnet.it

**Keywords:** military areas, pollution, Sardinia, ecotoxicologal effects, epidemiological data

## Abstract

This work assesses the environmental impact from chemical emissions due to military tests and routine activities in the area occupied by the Italian Inter-force Test Range (PISQ), located at Salto di Quirra, Sardinia, Italy. After reviewing the military activities carried out at PISQ, such as rocket launching, blasting and armament destruction, projectile and mortar fire impact, the associated pollution is evaluated. Chemical analyses were performed by means of Scanning Electronic Microscopy and Energy Dispersion Spectrometry on biotic and abiotic matrices. Residues of Rb, Tl, W, Ti and Al were found in matrices collected in the PISQ areas and environs. A review of experimental data on air, water, soil, milk, forage and animal tissues obtained by various Public Agencies of Sardinia proved that toxic element residues often exceeded the legal limits. PM_10_ and PM_2.5_ air concentrations also exceeded the legal limits after military blasting. Cd and Pb contents in the liver and kidneys of sheep living in farms at PISQ and in control farms that were located more than 20 km away from PISQ were higher than the legal limits. This work was performed to investigate concentration of xenobiotics in ecosystems emitted from PISQ activities. This assessment could be useful to focus future epidemiological studies carried out in PISQ and its neighbouring areas.

## 1. Environmental Pollution by Military Activities

Military activities have produced contaminated environments at many sites around the World. Public data reveal that environmentally hazardous military activities in USA have generated on average a ton of toxic waste per minute during the last eight years, making them the largest source of pollution in the USA [1]. The U.S. Army Corps of Engineers has stored waste coming from the production of nerve gas and pesticides during the Second World War and the Vietnam War era at the Rocky Mountain Arsenal in Denver (Colorado). This area and the Jefferson Proving Ground area in Madison (Indiana) are the most contaminated lands in USA, cordoned off because any remediation sis considered too dangerous to be carried out [2,3]. In the USA, 900 of the 1,300 most polluted sites—according to Environmental Protection Agency (EPA) assessment—are abandoned military bases or military industrial manufacturing and testing sites that produced weapons and military related products and services. These sites hazardous to human health and the environment include chemical warfare and research laboratories, training bases and abandoned disposal pits. Toxic elements and compounds found there include organic solvents, pesticides, machining oils, heavy metals, rocket propellants, diesel fuel, nuclear waste, unexploded ordnance, PCBs and toxic chemical compounds used in explosives. Until 1992 U.S. military bases were exempt from complying with U.S. environmental regulations. In 1995 the Department of Defence (DOD) identified 19,000 sites at 1,700 domestic military installations and more than 2,800 sites at 1,600 former defence facilities in the United States. Similar circumstances exist on foreign soil at the current or former U.S. military bases sites [4].

The Island of Vieques in Puerto Rico was used as a bombing range by the U.S. Armed Forces for about 60 years, and as a result many local residents were exposed to severe contamination. Epidemiological studies also suggest that certain illnesses are associated to the high environmental contamination in Vieques. Government health data indicated that risk of mortality from cancer was 1.39 times higher in Vieques than on the main island (Puerto Rico). Cancer risk in Vieques was found to increase since the early 70s [1].

In Tori Shima Island (Okinawa prefecture) one bombing range of the 38 U. S. military bases in Japan, the Marine Corps fired depleted uranium (DU) weapons in December 1995 and January 1996, although the use of DU weapons is banned in Japan. Military exercises in Okinawa have contributed to environmental damage by producing forest fires, soil erosion, pollution of surface waters and damaging effects on marine life, emission of toxic chemicals (PCB, Cd, Hg, Pb, *etc.*)—which are known to cause various health diseases—into the sea, extreme noise pollution, release of radionuclides by nuclear submarines [5,6,7].

At the Al Shifa Hospital (Gaza strip, Palestine) where 28% of total births in Gaza occurs, 27% of parents with birth defect children declared exposure to white phosphorous and 53% suffered home bombing and was involved in the removal of the resulting rubble [8]. Israel in January 2009 launched DU bombs that exploded on the heights above Gaza at the Israeli-Gaza border, near the Gaza shoreline and Mediterranean Sea. The onshore winds carried the radioactive dust and smoke over the border exposing the Israeli population to high levels of radiation in the air and producing permanent contamination of environment and food [9].

As reported by Magnone [10], several experiments with chemical and biological weapons were carried out on humans during the Cold War. The Ministry of Defence of USA reported in 2002 that in the years 1952–1971 pathogenic and toxic substances were diffused into the environment to investigate their effects on humans and ecosystems. Since 1942 military research programs were pursued in Camp Detrick Centre; production and test sites were located in Mississippi, Utah and Indiana. In 1949 large scale experiments were conducted on the New York population and in the San Francisco Bay area. Biological aerosols were introduced into the atmosphere in order to verify how many people could be infected (CD 22 Project). In the so-called Large Area Converge operation (1957) soils were contaminated with Zn and Cd compounds to evaluate the rate of diffusion of these elements. Israel has never subscribed to the Biological Weapon Convention (BWC); therefore, the Ministry of Defence of Israel has probably developed biological weapons programs, but the Israeli Government Agency has never reported any information on this matter.

According to Borzi and Crivelli [11], 239 tests on people were made in the years 1949–1969 to simulate biological attacks on some main US cities; for example on bus terminals of Washington D.C. and on the New York underground system. Porton Down is the UK research centre for chemical and biological weapons. In the Second World War years military scientists tested there the first biological weapons produced by Western nations so that German crops could be exposed to five million spore containers.

In Italy there are many territories where military activities and testing are carried out. The biggest one, located in Sardinia, is the Italian Inter-force Test Range based at Salto di Quirra, Sardinia, Italy, often referred to by Italian military authorities as Poligono Sperimentale di Addestramento Interforze del Salto di Quirra (PISQ). PISQ is located in the southeast of Sardinia, Ogliastra and Cagliari (Sarrabus-Gerrei) provinces (Italy) and is classified as a closed military zone by the Italian Armed Forces. Military activities have been carried out by armies of different countries during the last 50 years in different areas of PISQ. All activities that take place within its perimeter are covered by military secrecy, and for this reason, very few assays present new and original research.

PISQ provides technical and logistical support as part of the training of the multi-national military force. Since 2002 evidence of disease has been detected by the Local Health Units of the Italian Health Service [12], the Italian National Health Institute [12] and by the Epidemiology, Development and Environment Agency (ESA) [13], as reported by Bianchi [14] and Cocco [12]. Two veterinary surgeons of the Veterinary Health Service, G. Mellis and S. Lorrai [15], were charged by Local Health Units USL 4/Lanusei and USL 8/Cagliari of the Italian Health Service to investigate the health conditions of livestock and to develop a sampling and analysis plan for meat, milk and cheese samples from the PISQ territories.

The surveys inside and near the PISQ, point out a higher incidence of teratogenesis for animals and a higher cancer incidence for shepherds and workers in many different livestock farms, located near the sites where military activities took place, producing polluting emissions in air water and soil.

Some constraints associated to these epidemiological studies in this depopulated area have induced some researchers to examine in depth several ecotoxicological aspects [12,16]. Therefore, in this context researchers have been charged by the Italian Republic Prosecutor’s Office of Lanusei (Ogliastra Province, Sardinia) to carry out deeper investigations on the risks due to the human exposition to toxic elements and compound residues in biotic and abiotic matrices [17,18,19].

On the same island industrial activities also produce pollutant emissions which are potentially damaging to health and the environment. Several investigations were carried out by different authors in order to detect these effects. The main industrial activity in Sardinia has left some soil banks which represent environmental and health risks. Particularly the disused Baccu-Locci mine (Sarrabus-Gerrei in the south of PISQ) mainly exploited sulphides of arsenic and lead and also of copper, iron and zinc. Analytical contents of As, Cd, Pb and Zn in soil and sediment samples were found to be higher than the legal limits (Italian Republic D.M. 471/1999; tables A and B) [20].

Determination of blood cadmium was carried out on 265 human subjects living in a southwestern area of Sardinia (Sulcis Iglesiente) where coal mining and other industrial activities were localized. Individuals living near the industrial plants had geometric means (GM) of blood Cd concentration (0.79 μg/L) significantly higher than controls (0.47 μg/L) and residents of the mining sites (0.54 μg/L). Cd concentrations in blood are nevertheless strongly dependent on lifestyle and age factors. Smokers and older persons had much higher blood Cd levels [21].

Following the review of the impact of military activities in several countries and the description of general pollution sources in some Sardinia provinces, this study carries out an environmental impact assessment of military activities of PISQ. The main goal of this study is to assess the experimental data concerning toxic substance residues in biotic and abiotic matrices and their effects on terrestrial ecosystems within the PISQ area.

## 2. Materials and Methodologies

**Figure 1:**
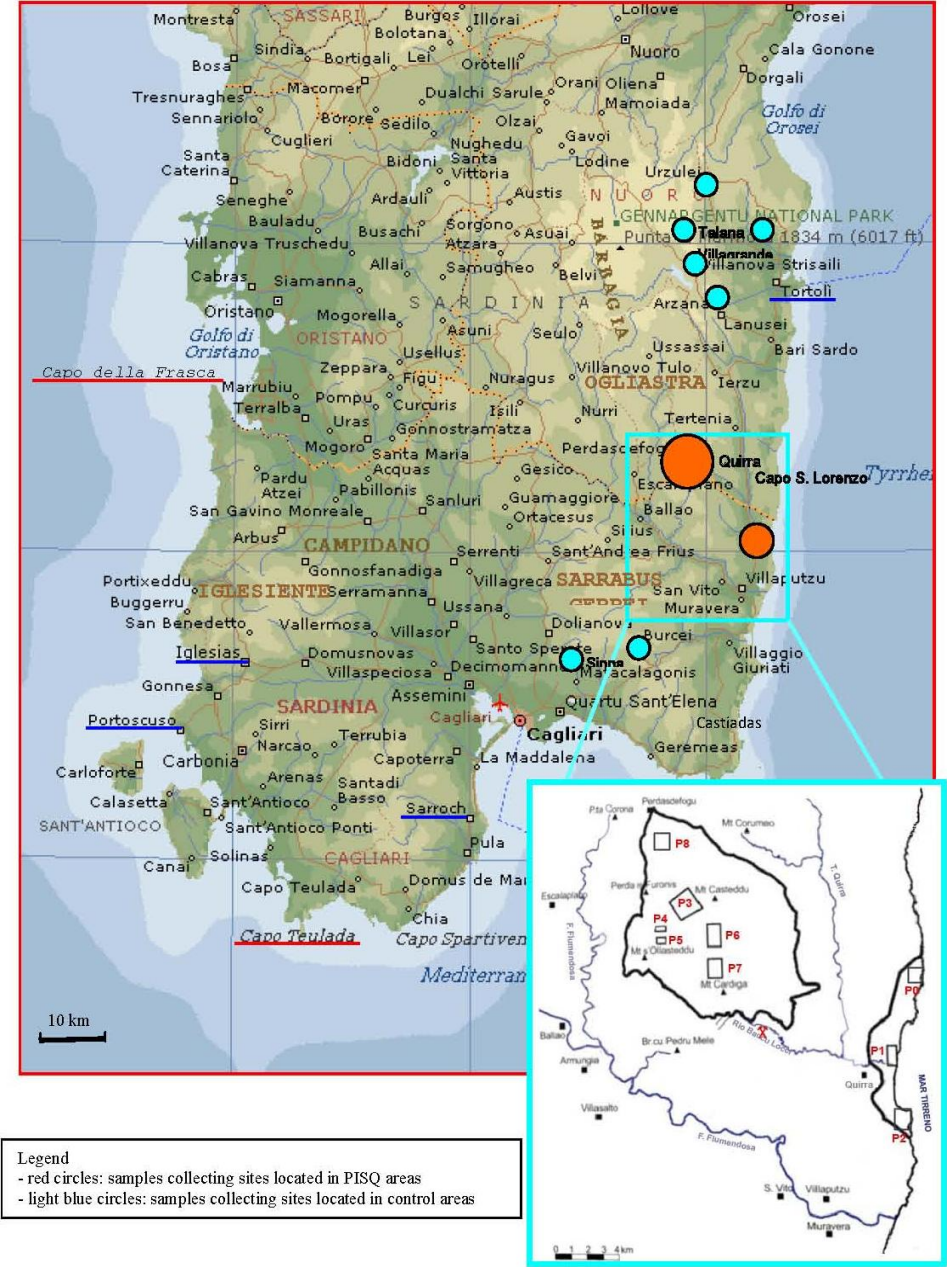
Geographic distribution of risk areas in Sardinia (Italy).

The PISQ area includes a land area (Perdasdefogu, Ogliastra) and a sea area (Capo S. Lorenzo, Sarrabus-Gerrei) (Figure 1). The following war games and military activities have been realized in the last 50 years in different areas of PISQ by armies of different countries (Figure 1):

1. Zefiro engine test-bed (VEGA rockets) (site: P0)
2. Hawk rocket launching (site: P1)
3. Nike rocket launching (site: P2)
4. Projectile and mortar fire impact area (site: P3)
5. Pressurization and fire ducts testing area (site: P4)
6. Blasting and armament disposal area (site: P5)
7. Tow and Milan rocket launching (site: P6)
8. Target shooting from helicopters (sites: P6 and P7)
9. Bullet impact on metallic armours (sites: P6 and P7)
10. Waste disposal of disused material (site: P8)

The chemical emissions associated to these activities are shown in Table 1 [22,23].

**Table 1:** Element and compound emissions by military exercises and testing [22,23].

Elements and Compounds	Test Type							
	Explosives	Ammunition Primers, Projectiles and Propellants	Rocket Propellants	Projectile Impact on Armour Metallic Surfaces	Blasting	Fire Bombs	Phosphourous Bombs	Tracer Bullets
Al	X		X					
Ag	X							
As	X	X						
Ba						X		
Be			X					
Cd	X		X		X			
Cr	X		X		X			
Cu	X	X		X	X			
Fe					X			
Hg	X	X			X			
Mg						X		X
Mn		X			X			
Ni			X		X			
Pb	X	X	X	X	X			
Rb			X					
Sb	X	X		X	X			
Si					X			
Sr								X
Ti				X				X
Tl					X			
V					X			
W				X	X			
Zn	X	X		X	X			
Zr						X		
CO	X		X		X			
CO_2_	X		X					
HBr			X					
HC (Hydrocarbons of kerosene)			X					
HCl	X	X	X		X			
Hydrazine			X					
NOx	X		X		X			
O_3_			X					
P_2_O_5_							X	
PAH					X			
PM_2.5_ (Particulate matter)	X		X	X	X	X	X	X
PM_10_ (Particulate matter)	X		X	X	X	X	X	X

The identification of elemental residues in inorganic samples, as well as biological samples, was carried out by Scanning Electronic Microscopy (SEM) at C.N.R. Molecular Genetic Institute (I.O.R., Bologna). The semi-quantitative analysis performed by SEM on samples collected in the areas in which different weapons were tried allowed the detection of dispersed toxic materials. In fact, this methodology allows us to identify residues of elements deposited in the boundary layers within samples, such as particulate matter emitted from military pollutant sources. The weight percentages of heavy metals and of other elements found on these samples surfaces, measured after a long time period from fall-out deposition, suggest that the multiple pollution from weapons persists in the environment, affecting living system. It is, therefore, evident that living systems such as plants, animals and men, can progressively accumulate the pollutants during their life cycle with possible genetic, somatic or epigenetic damages.

Samples were collected at farms in the following areas:

1. at “Casa Meloni” farm (Figure 1, red circle—Quirra, placed near P1 site) six pairs of samples of iron wire, concrete, soil, stone, roof concrete and lichens have been collected;
2. at “Casale Vacca” farm (Figure 1, red circle—Capo S. Lorenzo, placed near P2 site) seven pairs of samples of stone (taken in different points), stone and soil (taken in different points), lichen, chicken bone and lichens from the roof;
3. at “Is Pibiris” site (Figure 1, red circle—Perdasdefogu, placed near P8 site) two pairs of stone and lichen samples;
4. at the “St. Barbara” site (roadman’s house Km 81,462 state highway “Orientale Sarda”—CSL—placed near P1 site).

Every sample was picked up with tweezers and stored in plastic bags until the microscopy analysis in the lab. Samples were prepared under sterile lab conditions for scanning electron microscopy observations. Small pieces or fragments were selected and placed in an aluminum bowl, covered by silver coating and dehydrated. Every sample was observed and analyzed by microanalysis according to the following methodology: the biological samples were fixed in 2.5% glutaraldehyde in 0.1 M cacodylate buffer pH 7.4 for 3 h, post-fixed with 1% osmium tetroxide and dehydrated in a graded series of ethanol solutions. The inorganic samples, like the biological samples, were critically point dried and gold sputtered with an Edwards S150B apparatus and observed with a Cambridge Stereoscan 200 electron microscope operated at 20 kV. The microelement analysis was performed by Energy dispersive spectroscopy (EDS). The analysis was performed at 25 mm WD with an Oxford INCA Energy 200 apparatus.

## 3. Experimental Results

SEM indicated the presence of elements originating from both natural background and anthropic activities at the “St. Barbara” site. Considering the latter, the following elements were found:

1. Rb, Tl, W in Barn-owl (*Tyto alba*) pellets;
2. Rb and W in lichen samples;
3. Rb and W in some rock samples and in ground samples.

At the site “Casale Vacca” farm the following elements were found:

1. Al and Tl in some rock samples;
2. Al and Tl in some ground samples;
3. Al and Tl in some ground samples from a sheep pathway inside the farm;
4. Al and S in lichen samples.

At the other “Casa Meloni” farm Ti was found in some ground samples collected near sheep folds. At the “Is Pibiris” site Rb and W were found in stone and lichen samples. These elements are associated to chemical emissions produced by military experiments at PISQ. Rb residues found by means of SEM and EDS analytical methodology probably derived from the following military applications: electronic warfare, telemetry and the linked atomic clock used to improve missile guidance.

W content in surface matter of ground, rocks and lichens in the “St. Barbara” site were lightly diffused through particulate emissions, probably due to blasting coming from the “Perda Maiori” site (Figure 1, red circle—Perdasdefogu, located near the P5 site).

Tl found in both “St. Barbara” and “Casale Vacca” samples could have fallen on the surface after blasting activity and electronic components from launching rockets which emit oxides or sulphates of this element.

For what concerns the “Casale Vacca” site, analytical results of ground rock surface matter and of lichen tissues show also Al contents. This element is a chemical component of explosives and solid rocket propellants (Table 1).

Ti found in ground samples of the sheep fold at “Casa Meloni” could be particulate matter developed in blasting activities and also from gaseous emissions resulting from tests of projectile impacts on metallic armour surfaces.

The abovementioned experimental data should be compared with other analytical results carried out in the same area of PISQ and environs by several agencies and private laboratories, as reported by ARPAS (Environmental Protection Agency of Sardinia) and IZS (Experimental Zooprophylactic Institute of Sardinia “G. Pegreffi”). The analytical data concerning toxic element concentration in soils [16] are shown in Table 2.

**Table 2:** Toxic element residues in soils [16].

Pisq and Surrounding Areas	Toxic elements exceeding law concentration limits in soil ^1^
Area of Intense Activity—Perdasdefogu Range Military	As, Tl and less frequently: Cd, Co, Cr, Cu, Pb, Sb, Zn.
Neighboring Intense Activity Areas—Perdasdefogu Range Military	As, Tl and less frequently: Cd, Co, Pb, Sb, Zn.
Area of Intense Activity—Capo S. Lorenzo Range Military	As, Tl and less frequently: Cd, Cu, Zn.
Neighboring Intense Activity Areas—Capo S. Lorenzo Range Military	As, Tl and less frequently: Cd, Zn.
Civil Areas	As, Tl and less frequently: Cd, Co, Cr, Cu, Ni, Pb, Sb, Zn.

^1^ Enclosure 5, Title V D. Lgs. 152/06 (Norme In Materia Ambientale) Concerning Law Limit Concentration About Soil Contamination of Green Areas or Industrial Areas.

The main sources that generate these emissions are reported in Table 1. Tl residues in soil probably come from the glasses transparent to infrared radiation used in rockets or from some explosives.

## 4. Available Information about Health and Environmental Impact in PISQ

### 4.1. General Survey on Chemical Data in Biotic and Abiotic Matrices

In 2008 the Italian Ministero della Difesa (Ministry of Defence) financed with 2,500,000 euro the environmental monitoring of the PISQ and entrusted NATO Maintenance and Supply Agency (NAMSA, then named Nato Support Agency or NSPA) to realize it. Agreement was reached on five research lines in order to: (1) monitor radioactive pollutants diffusion in atmosphere; (2) investigate electromagnetic pollution; (3) carry out a chemical analysis plan of toxic elements and compounds on abiotic and biological matrices; (4) implement the ISO 14001 environmental certification; (5) develop the corresponding Environmental Information System (EIS).

The research line concerning the environmental monitoring and the health surveillance on humans and livestock animals was carried out by chemical and physical determinations during 2010 by private and public (ARPAS and IZS) laboratories.

Both analytical results concerning samples collected in the areas shown in Figure 1 (red and light blue circles) are not affected by emissions coming from neighbouring industrial and mining activities. Particularly, although the disused Baccu-Locci mine has produced heavy metals, it did not affect the sampling area (red circles in Figure 1) [20]. Moreover, other collection sites (light blue circles in Figure 1) that is, near Tortolì, the Intermare Arbatax Yard (Saipem Energy Service) don’t produce air pollutant emissions containing the toxic elements found in the samples analyzed by IZS and ARPAS. 

As reported by ARPAS [16], toxic organic and inorganic compounds are found in soil samples collected in Perdasdefogu and Capo San Lorenzo areas, and some civil sites in the same territory (only perchlorates):

1. Perchlorates (oxidant rocket solid propellants);
2. Trinitrotoluene (TNT)—(explosive);
3. 2 Amino-4,6-DNT and 2,4-DNT (highly toxic ballistite additives);
4. Royal Demolition Explosive (RDX)—triazine;
5. High Melting Explosive (HMX)—tetrazocine;
6. Tetrile (explosive).

Air pollution was measured by mobile (Capo San Lorenzo and Perdasdefogu) and stationary (Perdasdefogu) analytical monitoring equipment [16]. Particulate PM_10_ and PM_2.5_ concentrations in the area increased during and after military tests, exceeding the legal limits. Particulate PM_10_ and PM_2.5_ in Perdasdefogu and Capo San Lorenzo areas have shown similar chemical-physical characteristics. During and following rocket tests in Capo San Lorenzo areas (P1–P2 in Figure 1) an increase of As, Al, Ba, P, Na and K particulate content was found, probably caused by solid propellant emissions. Therefore As concentration in air was 350 times higher than the legal limits during Hawk and other rocket launching. During and following blasting operations in the Perdasdefogu area (P3 in Figure 1) As, Cr and Ni air concentrations in particulate increases over five times the legal limits were found (Legislative Decree 155/10, that is the application of Directive 2008/50/EC of the European Parliament and of the Council of 21 May 2008, on ambient air quality and cleaner air for Europe**)** [16].

The following experimental results were obtained by the Experimental Zooprophylactic Institute of Sardinia [24] and concerns the matrices associated to livestock activities: water, forage and animal tissues (Table 3, Table 4).

**Table 3:** Toxic elements concentration in livestock water [24].

Livestock water sample	Toxic Element	Concentration (µg/L)	Law limits * (µg/L)
*PISQ AREA farms*			
A	Sb	20.31	5
B	Cd	7.15	5
	Pb	12.3	10
	Fe	535.3	200
	Mn	136.3	50
	Ni	79.3	20
C	Cd	10.92	5
D	As	17.39	10
E	As	11.16	10
F	As	12.68	10
G	As	13.67	10
*CONTROL AREA farms*			
H	Cd	47.28	5
	Ni	29.69	20

* Recommendation of European Commission (5 June 2001) about the Inspection program on animal feeding according to regulations reported in 95/53/CE.

In Table 3 the data on livestock water from the PISQ area and control areas are shown. In seven of 43 samples coming from PISQ area farms, toxic element concentrations exceeded the legal limits, while in only one sample on 43 coming from control areas the concentration of two elements exceeded these limits. These excesses were measured for Sb, Ni, Mn, Cd and As (also in a livestock farm considered as a “control”, sic!) that are relevant toxic elements. In livestock waters collected in some farms of Burcei (“control area”, sic!) uranium (U) residues and Mo residues were found in a farm at Villaputzu (Sarrabus-Gerrei-Ogliastra, close to the PISQ area) [24]. Therefore the extension of the areas named “control areas” should be revised.

**Table 4:** Toxic elements concentration in forage [24].

Forage sample	Element	Concentration (µg/kg)	Law limits * (µg/kg)
*PISQ AREA livestock farms*			
A	As	2 < [Conc] ≤ 3.92	2
B	As	2 < [Conc] ≤ 3.92	2
C	As	2 < [Conc] ≤ 3.92	2
D	As	2 < [Conc] ≤ 3.92	2
*CONTROL AREA livestock farm*			
E	Mn	299.5	100

* Recommendation of European Commission (5 June 2001) about the Inspection program on animal feeding according to regulations reported in 95/53/CE.

Concerning the element contents in forage (Table 4), only four of 49 samples collected from PISQ farms have shown As concentrations exceeding the European Commission (EC) advice concerning livestock feed. In one of 31 samples coming from the control farms of Burcei, only Mn concentration exceeded the EC advice for forage [24].

U residues were found in five samples at Villaputzu (PISQ area) and Thorium (Th) was detectable in many samples collected both in Villaputzu and Burcei (about 20 km at South-SW of PISQ) [24].

Concentrations of dioxins, PCB, Pb and Cd in milk samples didn’t exceed the legal limits. However Th residues were measured in three milk samples in Burcei [24].

Considering Pb and Cd in liver, 14 of 27 samples collected from PISQ area farms have shown values exceeding the EC advice (Commission Regulation (EC) 466/2001 of 8 March 2001 setting maximum levels for certain contaminants in foodstuffs: liver content of Cd: 500 µg/kg; liver content of Pb: 500 µg/kg; kidney content of Cd: 1,000 µg/kg; kidney content of Pb: 1,000 µg/kg) only for Cd (max value found in samples: 1,455 µg/kg). Moreover in 20 samples out of 31 coming from control area farms, Cd concentration exceeded the EC advice (max value found in samples: 4,804 µg/kg).

Considering Pb and Cd in kidney, 25 of 27 collected samples from PISQ area farms have shown values exceeding the EC advice only for Cd (max value founded in samples: 16,410 µg/kg). Moreover, in all the samples except one (28 out of 29) coming from control area farms, Cd concentration exceeded the EC advice (max value found in samples: 21,280 µg/kg). 

Regarding muscle tissues, only in one sample of the 24 analysed, did the Cd concentration exceed the legal limit (20 µg/kg), reaching a maximum value of 147 µg/kg.

Within the PISQ Biomonitoring Project [16] 29 samples have been collected from several livestock farms located in the PISQ areas of Perdasdefogu (PDF) and Capo San Lorenzo (CSL). Average values of Cd concentrations exceeded the legal limits for kidney (12,400 µg/kg in PDF and 8,900 µg/kg in CSL) and in liver (1,720 µg/kg in PDF and 2,200 µg/kg in CSL) tissues. Only in samples collected in CSL, did Pb concentrations in liver (600 µg/kg) exceed the legal limits.

Among the toxic elements and compounds mentioned above in the experimental results some of these could be associated to cancer diseases and teratogenesis:

1. As: the skin is the main target of chronic exposure to organic arsenic, with the possible occurrence of skin cancer. Other adverse effects include liver damage, development of diabetes mellitus, peripheral neuropathy, peripheral vascular disease and internal cancers (bladder, lung, kidney and prostate) [25];
2. Cd: cadmium is classified as a probable human carcinogen (EPA: Class B; IARC: class 2A). A positive linear correlation has been observed between Cd concentrations in industrial environments and lung cancer, while chronic exposure to lower levels of Cd can lead to emphysema, bronchitis, heart disease, weakness of the skeletal system, anemia, depression of the immune system, kidney and liver pathologies [26];
3. Cr: chromium VI can cause damage to the liver and kidneys, internal bleeding, dermatitis and lung cancer. Exposure through inhalation causes nasal septum perforation, ulceration of the nasal mucous membranes and skin, and allergic dermatitis. The experimental data on animals exposed to Cr VI shows that the element has mutagenic and teratogenic properties. It’s classified as human carcinogen (EPA: Class A and IARC: Class 1) [26];
4. Ni: intake of this element is mostly through the gastrointestinal tract, but the most serious health damages are produced on the respiratory apparatus as lung cancer, obstructive respiratory pathologies, asthma and allergies, and on skin (dermatitis). Ni is classified as carcinogenic element of class A by EPA and class 1 AIRC [26].
5. Tl: this highly toxic element shows specific symptoms: hepatic necrosis, nephritis, central nervous system degeneration and teratogenesis affecting bone and cartilage [25];
6. 2,4-Dinitrotoluene (DNT) doesn’t exist in Nature. It is used to produce explosives, ammunition and dyes. DNT reduces sperm production in exposed workers and leads to a reduction in the number of red blood cells to nervous system, liver and kidneys disorders. DNT is classified as Class 2B “possible human carcinogen” by IARC [26].

### 4.2. Health Impact Assessment

The Italian Defence Ministry charged G. Mellis and S. Lorrai of the Veterinary Health Service of ASL 8 (Local Health Unit number 8) to investigate health conditions of livestock and to analyze various biological matrices (anellids, sheep, cheese, honey) [15]. The surveys inside and near the PISQ, pointed out a higher teratogenesis incidence for animals and a higher cancer incidence for shepherds and workers in many livestock farms (in comparison to regional data), located near polluting sources of air, water and soil generated by military activities.

Considering the epidemiological studies mortality (range period: 1997–2001) and hospitalization (approximately evaluated as mobility) data (range period: 2001–2003) for ten towns in which 26200 inhabitants live (Armungia, Ballao, Castiadas (a town is 30 kilometers further south from Quirra territory), Escalaplano, Muravera, Perdasdefogu, San Vito, Tertenia, Villaputzu, Villasalto (other municipalities sited in the same territory, like Jerzu, are not included), were studied by Biggeri *et al.* [13]. The area of these municipalities suffers the impact, although to different degrees, of PISQ pollutant emissions. Excess mortality due to haemolymphopoietic cancers, compared with regional data for Sardinia, was reported as +28% for male and as +12% for female individuals. In the same territories, considering hospitalization data, males show an excess of 10% for all cancers and also an excess of 65% (12% considering hospitalization for all diagnosis) for haemolymphopoietic cancers [12]. Female show an excess of 12% for haemolymphopoietic cancers.

Analyzing hospitalization data for diabetes, higher excess data are found for males (210%) and females (264%). Moreover, the excess of diabetes considering hospitalization for all diagnosis were respectively 58 % and 71% for males and females [13]. In Puerto Rico, where the Vieques military base is located, very high rates of diabetes were also observed (25% of the population has diabetes), Moret states that uranium interacts with the biochemical mechanism of insulin at a cellular level and damages the pancreas [27]. Kouznetsova *et al.* [28] found that residential proximity to persistent organic pollutant (POP)-contaminated waste sites results in increased rates of hospitalization for diabetes.

According to ASL 8 of Cagliari, it’s important to note that 90% of mortality caused by non-Hodgkin lymphoma is concentrated in the Muravera, San Vito and Villaputzu villages in which only 19.12% of total population considered in the epidemiological research live. Moreover in these villages lymphoemathopoietic cancer mortality accounts for 75% of the whole population studied and particularly, 40% in Villaputzu [29].

## 5. Tentative Conclusions

Elements and compounds were detected in several abiotic and biotic matrices associated with anthropic activities, likely military ones. Experimental evidence was found in air, water, soil and in animal tissues indicating that concentrations often are higher than the legal limits. 

Investigations carried out on animals and humans seem to indicate mortality and morbidity due to exposition to chemical xenobiotics. Exposures of people that live in the abovementioned villages of the PISQ-surrounding territories through air, water and food, could be higher than in other municipalities.

According to Burgio [30] some heavy metals could be carcinogenic agents. For this reason a complete assessment should be carried out using the data concerning chemical residues in abiotic and biotic matrices shown in the tables above. Besides, health impact assessments should include the exposure to electromagnetic non-ionizing radiation of people living in PISQ area, where we find 36 emission sources through military radar equipment according to Marinelli [31].

The excess of haemolymphopoietic cancers assessed in Sarrabus-Gerrei-Ogliastra territory (Villaputzu village) and the excess of congenital anomalies in Escalaplano town didn’t produce in past years any surveillance program of public institutions in this territory [12,14].

Particular analysis should be made on the Quirra hamlets (Villaputzu municipality), to identify specific clusters in small areas which are extremely significant to epidemiological research on the PISQ territories [32]. There are clinical reports of 168 deaths for cancer with detailed information collected by the Italian Republic Prosecutor’s Office of Lanusei, that should be deeply studied through epidemiological methods [33].

The follow up of these scientific evidences should be achieved. Investigations to identify the possible specific causes of mortality and excess hospitalizations should be made in these sites where high environmental pressure is recognized. Chemico-physical and toxicological characterization of exposure sources could facilitate epidemiological studies of clusters around these sources.

Ecotoxicological investigations should be developed in areas outside military sites where a diffusion of pollutant chemical elements and molecules is suspected. Recommendations should be advanced in order to not consider these territories as “negative control areas”.

The investigation should also be extended to the marine ecosystem inside the PISQ in order to produce any recommendations, as there is a small amount of data on it. Military pollution sources produce in fact emissions in air and water that could diffuse into the sea determining toxicological effects in biotic matrices and in food chain causing danger to human health.

Moreover, the reduction of biodiversity which has been observed in another research line in the PISQ territory, should be a suggestion to develop an investigation on the addictive or/and synergic effects caused by both xenobiotic exposures and climate change.

We also agree with the general recommendations of Commissione Tecnica Mista di Esperti [16] to:

1. Study wild and game animals, their products and by-products as sentinels of environmental hazards;
2. Study the bioavailability of the pollutants analysed;
3. Assess the impact of toxic elements and compounds on the food chain.

Finally, the knowledge of xenobiotic chemicals emitted by specific military activities in the last decades—considering different materials, processes and devices used (Table 1)—is useful to determine possible correlations with experimental concentrations found in the analyzed matrices.

Therefore, assessment of toxicological effects of these elements and compounds and health risks associated should be defined. The evaluation obtained should be correlated within the epidemiological studies carried out in the same areas.
